# Impact of Nutrient Intake on Hydration Biomarkers Following Exercise and Rehydration Using a Clustering-Based Approach

**DOI:** 10.3390/nu12051276

**Published:** 2020-04-30

**Authors:** Colleen X. Muñoz, Evan C. Johnson, Laura J. Kunces, Amy L. McKenzie, Michael Wininger, Cory L. Butts, Aaron Caldwell, Adam Seal, Brendon P. McDermott, Jakob Vingren, Abigail T. Colburn, Skylar S. Wright, Virgilio Lopez III, Lawrence E. Armstrong, Elaine C. Lee

**Affiliations:** 1 Department of Health Sciences, University of Hartford, West Hartford, CT 06117, USA; Wininger@hartford.edu; 2 Division of Kinesiology & Health, University of Wyoming, Laramie, WY 82071, USA; evan.johnson@uwyo.edu; 3 Onegevity Health, New York, NY 10019, USA; lkunces@gmail.com; 4 Virta Health, San Francisco, CA 94105, USA; Amylmckenzie@gmail.com; 5 Yale School of Public Health, New Haven, CT 06511, USA; 6 Department of Veterans Affairs, West Haven, CT 06516, USA; 7 Department of Health Promotion & Human Performance Weber State University, University of Arkansas, Fayetteville, AR 72701, USA; Corybutts@weber.edu (C.L.B.); arcaldwell49@gmail.com (A.C.); adseal@calpoly.edu (A.S.); Brendonm@uark.edu (B.P.M.); 8 California Polytechnic State University, San Luis Obispo, CA 93407, USA; 9 Department of Biological Sciences, University of North Texas, Denton, TX 76203, USA; Jakob.Vingren@unt.edu; 10 Department of Kinesiology, Human Performance Laboratory, University of Connecticut, Storrs, CT 06269, USA; atcolbur@asu.edu (A.T.C.); Skylar.wright@uconn.edu (S.S.W.); uconnla@aim.com (L.E.A.); elaine.c.lee@uconn.edu (E.C.L.)

**Keywords:** hydration, nutrition, sport nutrition, exercise, copeptin, collinearity, clustering

## Abstract

We investigated the impact of nutrient intake on hydration biomarkers in cyclists before and after a 161 km ride, including one hour after a 650 mL water bolus consumed post-ride. To control for multicollinearity, we chose a clustering-based, machine learning statistical approach. Five hydration biomarkers (urine color, urine specific gravity, plasma osmolality, plasma copeptin, and body mass change) were configured as raw- and percent change. Linear regressions were used to test for associations between hydration markers and eight predictor terms derived from 19 nutrients merged into a reduced-dimensionality dataset through serial k-means clustering. Most predictor groups showed significant association with at least one hydration biomarker: (1) Glycemic Load + Carbohydrates + Sodium, (2) Protein + Fat + Zinc, (3) Magnesium + Calcium, (4) Pinitol, (5) Caffeine, (6) Fiber + Betaine, and (7) Water; potassium + three polyols, and mannitol + sorbitol showed no significant associations with any hydration biomarker. All five hydration biomarkers were associated with at least one nutrient predictor in at least one configuration. We conclude that in a real-life scenario, some nutrients may serve as mediators of body water, and urine-specific hydration biomarkers may be more responsive to nutrient intake than measures derived from plasma or body mass.

## 1. Introduction

There is substantial and increasing interest in identifying foodstuffs that mediate or moderate body water [1,2,3,4,5]. This topic is particularly relevant to exercisers and especially to competitive endurance athletes whose activities typically last for multiple hours, requiring deliberate management of food and fluid intake. Several investigations have reported typical intake of carbohydrate, protein, fat, caffeine, and sodium of endurance cyclists on event day specifically, and note greater intake of carbohydrate than fat and greater fat than protein [6,7,8,9], modest intake of caffeine (approximately that of 1–2 cups of coffee) [7,8], and frequently high but quite variable intake of sodium (ranging from 852 to 8400 mg) [6,7,8]. Synergistic nutritional strategies become increasingly important when both the event and the environment are extreme [10,11]. Since endurance event organizers typically provide ample water and sports drinks or facilitate ample opportunities for participants to furnish and replenish their own supplies, access to fluids is rarely an issue. As a consequence, tactics shift away from fluid supply to strategic leverage of consumption, i.e., identification of food-fluid pairings that yield optimal water balance.

Many have investigated the role of beverage composition on hydration biomarkers (to represent hydration status), and while they often examine beverages with multiple nutrient composition, insight into the ability of isolated nutrients to successfully impact rehydration exists. Beverages that include sodium, particularly in concentrations greater than that lost in the sweat, effectively reduce urine volume and restore body water balance [3,12,13]. Notably, sports beverages typically contain less sodium than that lost in the sweat, generating great value in alternative means of sodium intake (i.e., foods and tablets). Carbohydrate inclusion in rehydration beverages consistently demonstrates the ability to improve body water balance following water losses, but could result in less total water consumption due to potential side effects such as bloating [14]. The addition of potassium [15,16,17] and protein [18,19,20] in beverages to rehydrate or promote body water balance has produced equivocal results, likely due to variable inclusion of additional nutrients (i.e., chloride with potassium, and the provision of milk to evaluate the effect of protein, which also contains carbohydrate, sodium and potassium). Collectively, previous works imply value in a broad inclusion of nutrient intake to maintain or restore body water balance, and athletes and exercisers tend to practice the intake of numerous nutrients through beverages, foods, and supplements. With more advanced statistical approaches, favorable sports nutrition practices to optimize hydration, performance, and health could be better substantiated.

In this field study, our primary objective was to measure the associations between nutrients we intentionally selected that are critical to osmotic homeostasis (Table 1) and biomarkers of hydration status. We expected that some nutrients might be consumed concomitantly among participants, and this circumstance creates a co-linearity that violates a basic assumption of linear regression and could lead to erroneous conclusions. Thus, we sought to identify individual nutrients that may be merged into a composite predictor variable, i.e., clusters of nutrients exhibiting consistent patterning within participants: two nutrients would cluster together if, e.g., when participants consumed a lot of nutrient A, they also consumed a lot of nutrient B (and likewise: a little of A corresponded to a little of B). Furthermore, hydration biomarkers would cluster together if they were similarly altered by practices during an endurance event. Ultimately, these considerations lead us to the following objectives: (1) to determine nutrient intake clusters (i.e., which nutrients were ingested in similar amounts), (2) to determine hydration biomarker clusters (i.e., which hydration biomarkers changed similarly during the event), and (3) how nutrient intake clusters influenced hydration biomarkers. We hypothesized that: (1) nutrients will cluster similarly to ingestion volume reported in previous literature (i.e., macronutrients would not, while sodium and carbohydrates would cluster together), (2) hydration biomarkers will cluster according to sample type (e.g., plasma versus urine), and (3) nutrients functionally related to osmotic homeostasis will relate to hydration biomarkers if acute consumption (i.e., electrolytes) affects hydration state. Additionally, we included analysis of plasma copeptin, which could serve as a putative arginine vasopressin (AVP) surrogate [21,22] and potential hydration biomarker [21,22,23,24,25] to determine whether there is evidence in this field study that copeptin may serve as a biomarker of hydration state. We hypothesize that if copeptin is a biomarker, it will be related to water and other hydration biomarkers. Because multicollinearity of independent variables can overinflate standard errors in traditional modeling and erroneously suggest insignificant relationships, our clustering-based, machine learning approach is novel and more appropriate for examining the relationship between nutrient intake and hydration biomarkers.

## 2. Materials and Methods

### 2.1. Participants and Conditions

The participant pool comprised cyclists registered for the Hotter’N Hell Hundred (HHH), a 161 km road bicycle ride held annually in Wichita Falls, Texas, in late August. The 2017 event drew 7414 participants who started their ride at 07:00 on 26 August 2017. The weather profile for Wichita Falls, TX, on event day was a low temperature of 21 °C, high temperature of 31 °C, zero precipitation, and dew point ranging from 20 to 22 °C. Wind speeds were low (peak 22.5 km⋅h^−1^, but predominantly below 16 km⋅h^−1^), Astronomical Twilight spanned 5.37 a.m. to 21.37 p.m. 

### 2.2. Recruitment

Cyclists were recruited via email and by personal approach on the two days prior to the ride at the event registration venue. Once in person at the event location, all participants provided written informed consent under procedures approved by the University of North Texas Institutional Review Board. Cyclists were eligible to participate in the study if they were 18 years or older, had previously completed at least one 161 km cycling ride, did not have a condition or were taking a medication that alters body fluid balance, purposefully omit one nutrient or class of nutrients from their diet (i.e., vegetarian, ketogenic diet), and do not use tobacco products. Participants were oriented to the study objectives, and advised that they would be queried for nutritional intake starting from anything consumed after PRE measurements, throughout the race, and upon their completion of the course.

### 2.3. Data Collection

Standard demographic variables were collected one to two days prior to the event, on site, including age, sex, height and weight. A Registered Dietitian (R.D.) and investigators trained by the R.D. interviewed each subject immediately following the event. Fluid intake was estimated by whole and fractional bottle amounts in addition to water contained in foods; gels and solid foods were accounted for by packaging (e.g., wrappers) when possible or self-report when necessary. Cyclists were also provided with small rubber bands that were placed on their handle bars, which were to be removed one at a time upon the consumption of an entire water/beverage bottle. Further, cyclists were informed upon recruitment that they would not be permitted to pour water from their water bottles over their bodies for cooling. The R.D. converted food consumption to nutrient weight (and Glycemic Load) via Nutritional Data System for Research (NDSR) software (Nutrition Coordinating Center, University of Minnesota, Minneapolis, Minnesota). Blood, urine and body mass were collected within 120 min of event start (“PRE”) and within ~10 min of event completion (“POST”); other than that provided as part of this study (described below), no food or fluid consumption was permitted following the event until completion of study procedures. To facilitate rapid collection of variables after the event, our research tent was placed at the finish line, we had three toilets dedicated solely for our use, we arranged stations for each variable (i.e., separate station for urine, blood and body mass collection), and cyclists were sent to stations that had availability as long as this didn’t interfere with critical measurement order (i.e., timing of urine and body mass measurements).

### 2.4. Water Bolus Intervention

While our principal interest was in measuring the relationship between nutrient intake and hydration status appertaining to the endurance event itself, there is additional perspective to be gained in assessing the body’s response to a hydration bolus immediately post-event: many athletes attempt to recapture optimal body water balance following their performance. Thus, following measurements at POST, study participants consumed 650 mL of ambient temperature water divided in six equal parts within three minutes. “Chugging” of the entire bolus was prohibited in an effort to repress body water regulation processes influenced by large bolus consumption [46] and even distribution was encouraged via instruction to consume one of the six parts every 30 s. During the hour following water bolus consumption, participants largely remained seated and were not permitted to void their bladder or bowel; upon one hour, they provided a third sample of blood and urine (“POST1h”), followed by body mass.

### 2.5. Data Conditioning

Each variable was inspected for normality via the Shapiro-Wilk test, in order to ensure that the assumptions of subsequent statistical analyses were upheld. Predictor variables meeting a pre-specified condition for non-normality (Shapiro-Wilk test result *p* < 0.05) were log-transformed to increase their normality; in order to protect the integrity of the log-transformation (i.e., so as to avoid undefined values resulting from log-0), we added 1 to all values prior to transformation. To further ensure the robustness of the analysis, outliers were removed by censoring the data from any single participant where any one nutrient value was extreme. Removal of the participant’s entire profile was intended to facilitate exploration without missing data. To ameliorate data loss and maintain ecological validity, the outlier threshold was set to four standard deviations beyond the mean. Data were then standardized (subtract the mean, and divide by the standard deviation) in order to facilitate scale-independent clustering, i.e., so that a nutrient, consumed in milligrams, did not obscure another nutrient consumed in grams (for example). Data were de-transformed prior to conducting all other analyses.

### 2.6. Nutrient Clustering

One of our main objectives in this work was to describe the association between a nutrient and a hydration-related response variable while accounting for collinearity via clustering. The 19 nutrient variables were grouped by k-means clustering with the number of allowable clusters ranging from 3 ≤ k ≤ 9. Because the k-means algorithm initiates cluster assignment by random selection, stochasticity was reduced by replicating the clustering 300 times per *k* (2100 replicates total; 7 k-levels × 300 replicates per level). An 18 × 18 co-clustering matrix C was initialized with all zeros; for each co-clustering of a variates *i* and *j*, C_ij_ and C_ji_ were incremented by 1. The final co-clustering matrix was normalized by 2100, so that 0 ≤ C_ij_ ≤ 1 for all *i* and *j*. Final co-clusterings were determined by inspection of two visual aids: (1) a heat-map-style correlation matrix showing elements of C as colored cells, and (2) a scatter in two-dimensions achieved through classical multi-dimensional scaling (MDS).

### 2.7. Hydration Biomarkers

Five hydration measures were collected: (1) percent body mass change (BMΔ), (2), urine specific gravity (U_sg_; Atago Inc., Model A300CL, Spartan, Tokyo, Japan), (3) urine color (U_col_) [47], (4) plasma osmolality (P_osm_; freezing point depression; Advanced Instruments, Model 3320, Norwood, MA, USA), and (5) plasma copeptin (P_cop_; Thermo Scientific BRAHMS Copeptin proAVP KRYPTOR). Body mass was measured at PRE, POST and POST1h, and therefore BMΔ was measured at POST and POST1h. The four measures related to urine or plasma were collected at all three time-points: PRE, POST, and POST1h. All U_sg_ and P_osm_ samples were measured in controlled environmental conditions (i.e., inside the event center building) soon after their collection. Selected outcome measures were reported as both raw value (measured at POST and POST1h), and also percent change (POST minus PRE, divided by PRE; and POST1h minus POST, divided by POST), yielding 16 reported hydration biomarker values. BMΔ was reported as raw value, adding two additional hydration biomarkers (18 total). All urine biomarkers were collected following bladder void; subjects who reported defecation during the ride or prior to study completion were not included in analyses.

### 2.8. Statistical Analysis

Hydration biomarkers were assessed for mean differences across time via One-Way Repeated Measures ANOVA or the Friedman test for normally and non-normally distributed data, respectively (whereas BMΔ was assessed via paired *t*-test due to its measurement at two versus three time points); in the event of a significant F statistic, Tukey’s or Dunn’s multiple comparisons tests were employed for normally and non-normally distributed data, respectively (Prism GraphPad 7).

Our interest was in describing the association between nutrients and select hydration markers via linear regression. By using nutrients averaged within a cluster, each cluster represents one or more nutrients, consumed in a distinct, consistent pattern across riders. Using the clustered nutrients, a series of standard linear regression analyses was performed in order to ascertain the nature of the association between consumed nutrients and various hydration markers. Where a nutrient-cluster yielded statistical significance against a hydration marker, the *p*-value and regressor coefficient were noted. For each analysis, participants with missing data were censored from the regression. In order to ensure the appropriateness of the regression model, each of the regression models was optimized via step-wise backward feature selection through minimization of the Akaike Information Criterion (AIC). The AIC is a standard measure of the tradeoff between model complexity (number of terms) and model goodness (residual error); the model with the lowest AIC is the optimal model.

Lastly, as an exploratory analysis, we separately assessed the relationships between hydration markers. This analysis used a similar approach to the clustering on nutrients, but more straight-forwardly: the 18 hydration markers were correlated against one another and viewed both as a hierarchically-clustered heatmap and as a two-dimensional scatter through MDS.

All cluster-based analyses were performed in the R numerical computing environment (R version 3.4.3).

## 3. Results

### 3.1. Descriptive Statistics and Changes in Hydration Status

**Figure 1:**
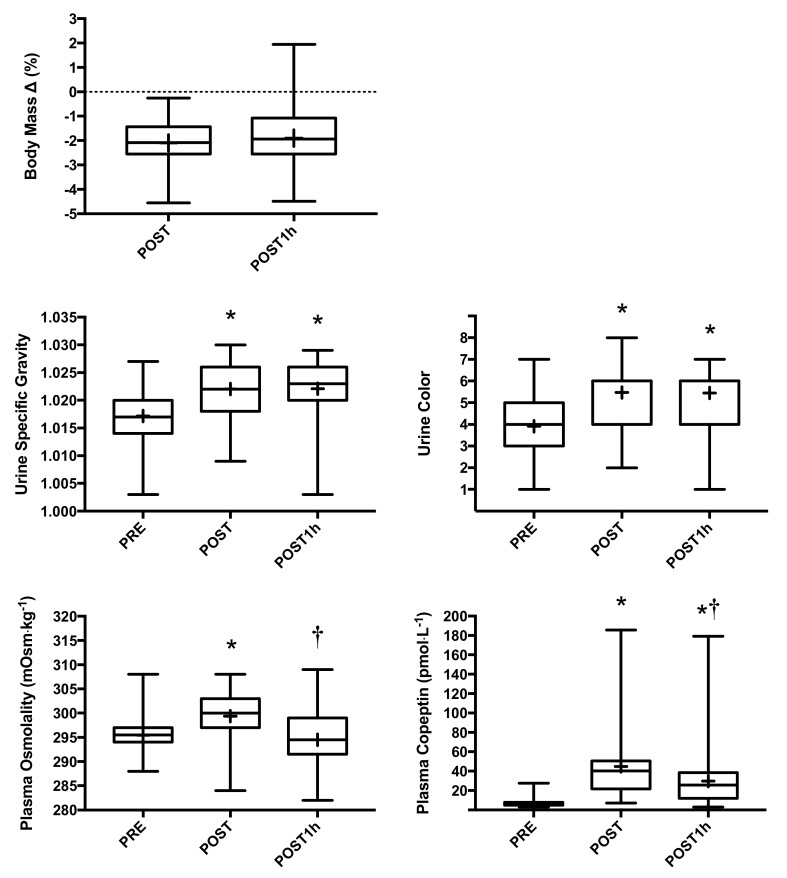
Summary statistics for hydration biomarkers before (PRE) and following the 161 km cycling ride (POST), and one hour after consuming the 650 mL water bolus (POST1h). * represents statistical difference from PRE and † from POST; + represents the time point mean.

Hydration biomarker and nutrient intake data were analyzed from 51 subjects. Four participants’ data were censored from analysis due to outlier data, resulting in a final sample size of 47 (mean ± SD unless otherwise noted; 44 males, 3 females, age = 52 ± 10 year (range = 21–72 year), height = 177.1 ± 6.1 cm, mass = 87.9 ± 13.4 kg, finish time = 374.9 ± 74.8 min). Via the Shapiro-Wilk normality test, 16 of 19 nutrients were found to be non-normal (all except Carbohydrates, Glycemic Load, and Water). Response variables contained 41 missing values among 714 data-points: 16 in Pre-Plasma osmolality, 7 each in Post-Plasma osmolality and Post-1h Plasma Osmolality, 6 in Post-Body Mass Loss, and 5 in 1hPost-Body Mass Loss; there were no missing data among 918 predictor variables. Summary statistics for each nutrient are reported in Table 2, and summary statistics and time point comparisons for hydration biomarkers are reported in Figure 1. Participants experienced mild hypohydration following the event according to the collection of hydration biomarkers. Consumption of the 650 mL water bolus (evaluated at the POST1h time point) did not impact BMΔ or urinary markers, and imply that participants remained in a hypohydrated state. In contrast, consumption of this bolus reduced P_osm_ to pre-ride levels and suggest that participants successfully rehydrated. P_cop_ identified a positive impact of the water bolus on hydration status but correctly maintained differentiation from PRE, since 650 mL of water intake was meaningfully less than the estimated amount of water lost during the ride (~1.7 L).

### 3.2. Nutrient Clustering

**Figure 2:**
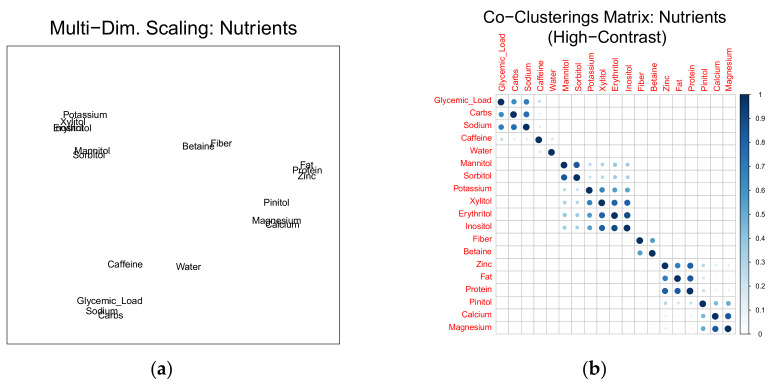
Graphical depictions of associations among 19 nutrients following serial k-means clustering. Multi-dimensional scaling view (**a**) and heat map of co-clusterings matrix (**b**); heat-map clarity enhanced by raising C_ij_ values to the 7th power.

The 19 predictor variables were serially clustered via k-means, over a range of k values. The co-clusterings (on a scale of 0 to 1, representing the proportion of 2100 clustering iterations where any two nutrient pairs were observed in the same cluster) were converted to a pseudo-distance matrix (1 minus co-clustering frequency) and plotted in two-dimensions via multi-dimensional scaling (Figure 2a). For further clarity, the co-clusterings matrix was inspected for the proportion of cluster replicates where nutrient pairs were co-clustered (Figure 2b). The co-clustering matrix represents the frequency of co-clustering among nutrients where a large dark circle indicates a co-clustering frequency near 100%, and a small light-blue circle indicates an infrequent co-clustering. In order to facilitate visualization and the process of manual extraction of clusterings among nutrients, this co-clustering matrix was raised to a large, odd-numbered power (set at 7), enhancing visual contrast.

**Figure 3:**
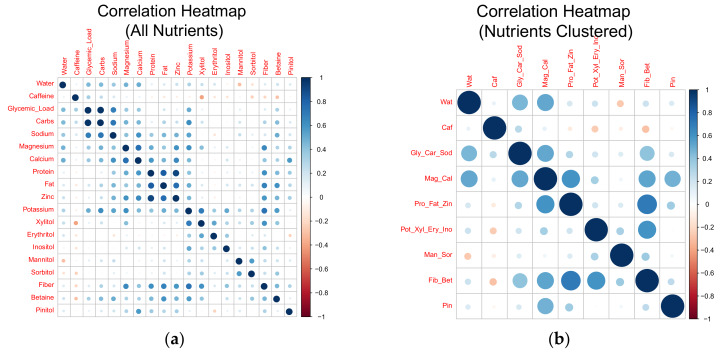
Correlation heat-map of 19 nutrients, hierarchically clustered (**a**) and consolidated through clustering and ensemble-averaged (**b**).

The two panels in Figure 2 were inspected for naturally occurring clusters. Nine clusters resulted: (1) Water, (2) Caffeine, (3) Glycemic Load + Carbohydrate + Sodium, (4) Magnesium + Calcium, (5) Protein + Fat + Zinc, (6) Potassium + Xylitol + Erythritol + Inositol, (7) Mannitol + Sorbitol, (8) Fiber + Betaine, and (9) Pinitol. The two panels in Figure 3 demonstrate second-order association, i.e., correlation between the nutrients before and after clustering.

### 3.3. Hydration Biomarker Clustering

**Figure 4:**
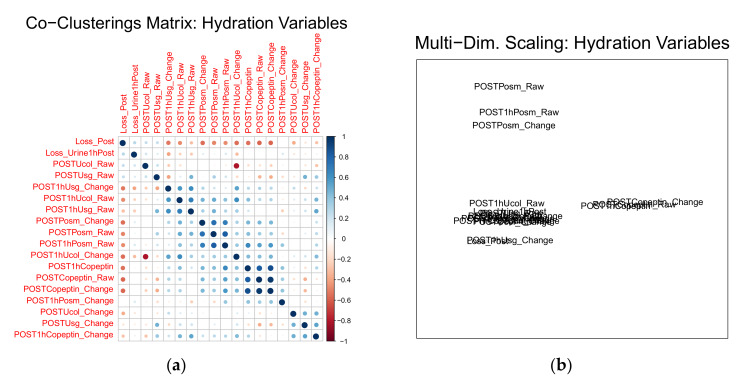
Graphical depictions of associations among 18 hydration biomarkers. Correlation heat-map (**a**); multi-dimensional scaling view (**b**); multi-dimensional scaling view created by raising correlation matrix to the fifth power.

With regard to the clustering of hydration biomarkers, correlation analysis provided ambiguous results (Figure 4a); however, multi-dimensional scaling revealed three clusters: (1) P_osm_, (2) P_cop_, and (3) all-other markers (Figure 4b). Within all four serialized measurements (U_col_, U_sg_, P_osm_, and P_cop_), all three time-points clustered together in the multi-dimensional scaling view.

### 3.4. Relationship between Nutrient Intake and Hydration Biomarkers

Table 3 shows the outcomes of the regression modeling. Empty cells indicate predictors that did not survive the sieve of the step-wise regression. For each of the five hydration biomarkers, at least one formulation (absolute versus percent change, or post- versus post1h minus pre) yielded at least one significant association to a nutrient cluster at the *p* < 0.05 level.

## 4. Discussion

This is the only study, known to the authors, to specifically address the expected nutrient collinearity through a clustering-by-homology approach, which may be a more appropriate approach. Through this, we have obtained a map of relationships between these nutrient variables based purely on inter-subject variability, based on departure from the group mean (Figure 2). We extend on our findings by showing a second-order association, i.e., correlation between the nutrients before and after clustering (Figure 3). Secondly, this is the first study to test the association between these clustered nutrients and a large number of hydration biomarkers, configured as both absolute and percent change. Thirdly, this is the first study to measure the relationships between four variations (absolute value and percent change, in immediate-post and post-1 h following a water bolus) of five different hydration biomarkers (two urine markers, two plasma markers, and body mass change; Table 3).

### 4.1. Primary Findings

1. Nutrient clusters formed analogously to intake patterns in previous studies: carbohydrate and sodium intake clustered together; however, fat and protein intake were similar and therefore clustered together. Most sugar alcohols clustered together, as did magnesium and calcium.
2. All urinary markers (regardless of time point) clustered together, while P_osm_ and P_cop_ remained independent of one another.
3. As expected, water itself and Mag + Cal were most frequently associated with hydration biomarkers among all 19 nutrient predictors; water tended to impact blood, while Mag + Cal tended to impact urine and body mass indices of hydration status.
4. Urinary biomarkers appear to be impacted by nutrient intake more than plasma biomarkers or body mass changes (calculated simply as % change from PRE).
5. Selection of absolute versus percent change measurement units for hydration biomarkers can differentially impact results.
6. P_cop_ utility as a hydration biomarker has promise but could be convoluted by the presence or cessation of exercise and potentially by nutrient consumption (i.e., Water and Pinitol).

### 4.2. Interpretation

Model fits were generally weak (*R*^2^ ≤ 0.37), suggesting that many factors (not dietary consumption alone) influence hydration biomarkers. Beyond water intake or hydration status, copeptin can also be influenced by heat exposure [25,48] and exercise [49], which limits its utility as a hydration biomarker. Similarly, heat exposure and exercise impacts P_osm_ [50] and urinary indices [51,52], which highlights and supports lack of a gold-standard tool for hydration assessment [53].

While some nutrients clustered as expected (i.e., carbohydrate and sodium intake), these cluster formations likely occurred (at least in part) due to common composition of products stemming from functional relationships known to the sport nutrition industry who seek to optimize function and health during exercise. For example, the electrolytes magnesium and calcium support water retention and have interdependent absorption and excretion processes [54], and magnesium also plays a noteworthy role in calcium transport to facilitate muscle contraction [55]. Total carbohydrate and glycemic load unsurprisingly formed a cluster, since glycemic load estimates the blood glucose response to net carbohydrate (i.e., carbohydrate minus fiber). From a functional perspective, glucose stimulates transport of sodium in the small intestine [56], and both are well known to enhance endurance performance via energy production and body water balance. Xylitol, erythritol, inositol, mannitol, sorbitol, pinitol and betaine serve as organic osmolytes but, of this group, betaine is the only nutrient not considered a polyol. Accordingly, it is not surprising that many of these short-chained carbohydrates were ingested in similar amounts since they are often ingested sparingly to avoid gastrointestinal distress [57]. The literature on betaine’s effectiveness as an ergogenic aid is equivocal [58], and therefore does not receive substantial focus in sports nutrition.

As anticipated, participants experienced negative body water balance at POST as supported by all hydration biomarkers measured, and urinary indices and P_cop_ but not P_osm_ implied continued need for fluid replacement following partial rehydration (POST1h; Figure 1). POST1h only examined partial rehydration, since 650 mL of water ingested by these cyclists equated to 56% of the 150% mass loss replacement recommendation following exercise-induced dehydration [11]. In consideration of how nutrient intake impacts these hydration biomarkers, we find it noteworthy that exercise does not appear to alter intestinal absorption of nutrients at or below intensities of approximately 70% VO_2_ max [59]; due to the duration of this event, it is unlikely that many of our participants exceeded this intensity or exceeded it for lengthy periods of time. The most significant findings between nutrient clusters and hydration biomarkers were observed in urinary versus plasma indicators or body mass change (Table 3). While previous investigations have only identified vitamin B2 as being capable of acutely darkening U_col_ independent of hydration status [60], we cannot rule out the possibility of U_col_ changes solely due to nutrient intake. Beyond these considerations, interpretation of urinary biomarkers during exercise comes with challenges: does more concentrated urine reflect insufficient or successful retention of water intake? We suspect the latter in accordance with long agreed upon previous work [61].

Water intake notably corresponded with reduced P_osm_ only at POST1h, and P_cop_ only at POST (with a trending association at POST1h). In isolation, water intake dilutes the extracellular compartment and induces neuroendocrine regulatory processes to tightly and rapidly control plasma concentration, and potentially urine constituents. We suspect that the significant inverse relationship between water intake and P_osm_ at POST1h reflects the recent 650mL of water consumption, and lack of relationship at POST reflects insufficient water intake during the event and/or hemoconcentration due to exercise. We also suspect that a lack of relationship between water intake and P_cop_ at POST1h signifies insufficient water intake to completely offset losses accrued during the event; in other words, perhaps P_cop_ indicates body water balance more so than P_osm_. The mismatched relationships between water intake and P_osm_ and P_cop_ challenges osmolality as a primary driving force for greater plasma copeptin, although transient alterations in P_osm_ would not have been detected in our investigation. While water intake, particularly at POST1h, did not consistently dilute urine in this investigation (Figure 1; Table 3), we speculate that in addition to reduced glomerular filtration rate due to prolonged exercise [62], water needs were great enough to prioritize retention over excretion, and these cyclists consumed sufficient dietary osmolar loads to aid in this retention.

Carbohydrates (particularly glucose) have long been reported to enhance absorption of sodium and water in the intestine [63]. Therefore, the collinearity of glycemic load, total carbohydrate, and sodium was unsurprising and likely reflects their regular incorporation into sports nutritional strategies. Our results suggest that carbohydrates, glycemic load and sodium collectively diluted the plasma and concentrated the urine, which implies this nutrient group aided in body water conservation and aligns with previous literature [27]. However, this nutrient cluster also corresponded with reduced body mass and implies greater body water losses via sweat. While we are unable to specifically explain this phenomenon, perhaps protecting P_osm_ permitted higher exercise intensity and therefore greater sweat losses. We also found that magnesium and calcium intake most meaningfully predicted reduced U_sg_ and greater BMΔ at POST, both of which indicate increased water losses despite the need to retain body water. Perhaps consumption of magnesium and calcium permitted reduced U_sg_ at POST due to: 1) ample time allotted for intracellular magnesium uptake (since magnesium is the second most abundant cation inside the cell), which then did not contribute to water retention of the extracellular compartment, and 2) a mild increase in plasma volume attributable to calcium [64], which could promote diuresis.

Upon initial consideration, the relationship between pinitol with U_col_ and P_cop_ appeared particularly interesting since pinitol serves as an organic osmolyte in plants [65] and is purported to enhance glycogen synthesis, which concomitantly stores water [66]. However, the very low quantities of pinitol intake observed in this investigation (0–0.028 g) seems unworthy of strong practical consideration since 2.5 g is considered a low dose of acute pinitol ingestion [67]. Intake of pinitol rich foods (such as soy and legumes) or supplements were not practiced in these cyclists, yet pinitol significantly predicted U_col_ at POST and P_cop_ at POST1h, which warrants additional investigation. While participants did not consume large quantities of caffeine (~65 mg), we did not find relationships between caffeine intake and any hydration biomarker. These data align with accumulating literature [1,45,68] and supports caffeine’s use as an ergogenic aid.

Practically, the regression coefficient (β) intends to inform the expected unit and directional change in the dependent variable according to the unit and directional change in the independent variable (i.e., every x mg of intake should result in x pmol⋅L^−1^ decrease in plasma copeptin). Due to the need to standardize and cluster the independent variables in this analysis (i.e., nutrients), it is not possible to determine the specific unit change in the nutrient cluster necessary to alter a particular hydration biomarker by a specific magnitude. Nonetheless, a more general interpretation of the magnitude of directional change in a particular hydration biomarker according to increased intake of a nutrient cluster maintains practical value. While model fits were largely weak, we attribute this to the numerous factors that contribute to body water balance and/or these particular biomarkers (i.e., exercise and environmental conditions).

### 4.3. Design Considerations

While research frequently seeks to induce drastic changes in hydration status in the investigation of biomarkers, this was not an objective in the current study. Because we aimed to examine relationships between nutrient intake and common hydration biomarkers, we hoped to and succeeded in observing cyclists who maintained (or nearly maintained) a euhydrated state and others who completed the event in a hypohydrated state. Those who maintained a euhydrated state consumed more water and other nutrients capable of retaining water consumed, and the directionality of our results align with this concept.

There is wide recognition that nutritional intake and hydration variables are substantially collinear and therefore ill-posed for direct integration into a regression-based model [69,70]. While approaches vary, there are some important limitations. For instance, Principal Components Analysis (PCA) confounds interpretation by allowing a single variable to be represented in multiple terms within the regression model; this is especially true in the scenario where two terms, carrying the same variable, yield opposite signs in the regression model. Nevertheless, the aspiration of PCA, i.e., to consolidate variability, is essential and underlies the approach used here.

Substantial thought was given to alternative approaches for the regression. Hierarchical regression was discarded for practical reasons: we disclaimed any expectation of which predictors might supersede which others in terms of predictive value, and the large number of variable combinations made exhaustive search computationally intractable. A linear mixed effects model with random effect on the pre-measurement value of each hydration marker was considered, but rejected for its circularity with the response variable, and due to the repeat nature of this study design (i.e., serial measurements throughout the event).

### 4.4. Limitations

Due to a software limitation, we were not able to account for chloride intake and therefore its impact on body water balance in this investigation. Additionally, our study took place in the setting of recreational athletes, immediately prior-to and immediately following a long duration exercise bout in a warm (mean = 26 °C, max = 30 °C) and humid (mean = 76% RH, max = 93% RH) setting. We note that the average Low and High temperatures in Dallas (nearest major metropolitan area, approximately 3 h away) in late August is typically 25 to 36 °C, respectively, with relative humidity ranging from 80% (morning) to 40% (afternoon). Thus, HHH-2017 was an atypically cool and moist day. We also speculate that nutrient intake timing (i.e., more or less water intake during the latter portions of the ride) could have impacted findings at POST. While this study gives some insight into the manipulation of nutrient intake and body water balance, our study is limited in its generalizability. However, there is much to be gained in studying athletes in moments of extreme conditions that cannot be captured by studying the general populace in more temperate conditions.

These data were collected in the field, and did not permit the calculation of real body mass change attributable to body water balance alone, which requires accounting for all mass losses (sweat, urine, feces, and that from glycogen water release) and gains (fluid and food intake as well as water generated from substrate oxidation). Due to great variation in some of these variables, a more extensive body mass loss calculation would have provided a vague estimation of actual water losses and would not have aligned with our attempt to provide a real-world look at the impact of nutrient intake on common hydration biomarkers (i.e., few athletes and exercisers incorporate extensive body mass calculations). Previous literature comparing more complex to simplified approaches of body mass loss as an indicator of hydration status found that simple post minus pre exercise body mass measurements were still useful estimates of determining body water changes, and that athletes would not conduct more complex calculations routinely [71]. While a controlled laboratory setting would surely allow measurement of the sources of variance experienced in our study, a field study makes possible the “massively parallel” data collection in a natural setting.

### 4.5. Practical Applications for Sport

In broad examination of Table 3, in particular, diet or supplement formulation for promoting hydration status should likely include water, carbohydrate, sodium, magnesium, calcium, and perhaps pinitol (pending further investigation). While many sport supplements and beverages include these components, these data could provide guidelines for those who wish to focus on consumption of foods versus (sometimes pricy) products from the sport nutrition industry. From a research perspective, we suggest implementation of our, or a similar, statistical approach in the study of multiple independent variables and to address practical implications.

### 4.6. Future Work

There are several aspects of this study which bear replication and extension by others. Firstly, given the modest sample size and relatively cool setting of the 2017 HHH, aggregation of data from additional years or additional events would add valuable depth to the dataset. Secondly, while we believe clustering is an attractive approach for its ability to consolidate collinear variables while avoiding the adverse scenario where a given nutrient is represented in multiple regression terms (i.e., Principle Component Analysis), we recognize the stochasticity inherent in k-means and the sensitivity of clustering algorithms in general to variable composition: adding or subtracting even a single nutrient may have substantial impact on the apparent co-clusterings, and thus on the regression models. We do not suggest that the present findings are definitive; rather, we acknowledge that they are cross-sectional and of unknown generalizability. We encourage others to replicate our approach and develop strong recommendations for promoting optimal hydration status through the aid of specific nutrient intake. Lastly, we suggest that by reporting co-associations between nutrients and among hydration markers, we are providing opportunity for others to revisit prior analyses. In cases where models were over-fit, or type-II error is suspected, reconfiguring the variables may provide new insight; where models were assumed to be appropriate, re-testing under a new variable configuration (where appropriate) may provide valuable validation.

## Figures and Tables

**Table 1:** Dietary nutrients related to osmotic homeostasis.

Nutrient Categories	Nutrients Available from Dietary Analysis	Support for Role in Osmotic Homeostasis
***Macronutrients*** ***(& related variables)***		
	Water	[11,26,27]
	Fat	[28]
	Protein	[4,29,30]
	Carbohydrate (& in theory, Glycemic Load)	[14,31,32,33,34]
	Fiber	[35,36]
***Electrolytes***		
	Calcium, Magnesium, Zinc, Sodium	[11,34,37,38]
***Organic Osmolytes***		
	Erythritol, Inositol, Mannitol, Pinitol, Sorbitol, Xylitol, Betaine	[39,40,41]
***Other***		
	Caffeine	[1,42,43,44,45]

**Table 2:** Summary statistics for nutrient intake the morning of and during the ride (mean (range)).

Nutrient Category	Specific Nutrient	Amount Consumed
***Macronutrients*** ***(& related variables)***		
	Water (g)	4468.3 (3644.3–5483.0)
	Fat (g)	22.3 (13.0–37.2)
	Protein (g)	26.9 (13.1–33.9)
	Carbohydrate (g)	268.6 (188.0–324.0)
	Glycemic Load	186.1 (113.6–254.4)
	Fiber (g)	11.2 (7.3–16.6)
***Electrolytes***		
	Calcium (mg)	485.6 (324.1–564.0)
	Magnesium (mg)	231.2 (157.0–295.5)
	Zinc (mg)	3.6 (2.2–4.9)
	Sodium (mg)	1690.0 (994.6–2454.3)
	Potassium (mg)	1190.1(869.7–1811.7)
***Organic Osmolytes***		
	Erythritol (g)	0 (0–0.001)
	Inositol (g)	0.16 (0.04–0.38)
	Mannitol (g)	0.003 (0–0.054)
	Pinitol (g)	0.01 (0–0.028)
	Sorbitol (g)	0.013 (0–0.072)
	Xylitol (g)	0.009 (0.003–0.0175)
	Betaine (mg)	26.7 (9.1–78.7)
***Other***		
	Caffeine (mg)	64.6 (5.3–124.4)

**Table 3:** Threshold *p*-values (*p*) and regression coefficients (β) for each nutrient cluster versus hydration biomarker in absolute (Abs) and percent change (%Δ) units. Model Fit reported as multiple-*R^2^*.

Biomarker		Body Mass		Urine Specific Gravity				Urine Color				Plasma Osmolality				Plasma Copeptin			
Time Point & Unit		POST	POST1h	POST		POST1h		POST		POST1h		POST		POST1h		POST		POST1h	
		% Δ	% Δ	Abs.	% Δ	Abs.	% Δ	Abs.	% Δ	Abs.	% Δ	Abs.	% Δ	Abs.	% Δ	Abs.	% Δ	Abs.	% Δ
Model Fit		0.22	—	0.13	0.30	0.32	0.19	0.28	0.37	0.08	0.07	0.07	—	0.35	0.20	0.13	0.05	0.29	0.26
Grp 1 Wat	*p*			0.071				0.032			0.142			0.006	0.002	0.014		0.09	
	*β*			−0.38				<|0.01|			−0.01			−14.3	−3.38	−10.45		−0.33	
Grp 2 Caf	*p*									0.069		0.099							
	*β*									−1.54		−1.43							
Grp 3 Gly + Carb + Sod	*p*	0.007		0.121	0.009		0.073	0.055						0.035					
	*β*	−0.61		0.36	0.16		<|0.01|	<|0.01|						−11.5					
Grp 4 Mag + Cal	*p*	0.002			<0.001	0.005			0.009									0.089	
	*β*	0.70			−0.25	<|0.01|			<|0.01|									0.34	
Grp 5 Pro + Fat + Zin	*p*			0.128		0.007		0.134											
	*β*			−0.31		<|0.01|		<|0.01|											
Grp 6 Pot + Xyl + Ery + Ino							0.133										0.115		
	*β*						<|0.01|										0.08		
Grp 7 Man + Sor	*p*																	0.107	0.093
	*β*																	0.28	0.33
Grp 8 Fib + Bet	*p*					0.009		0.173											0.166
	*β*					<|0.01|		<|0.01|											−0.30
Grp 9 Pin	*p*						0.153	0.049	0.016										0.002
	*β*						<|0.01|	<|0.01|	<|0.01|										0.54
